# Effects of feeding methods on growth and slaughter performance, blood biochemical indices, and intestinal morphology in Minxinan black rabbits (*Oryctolagus cuniculus*)

**DOI:** 10.1007/s11250-023-03467-w

**Published:** 2023-01-28

**Authors:** DongJin Chen, ShiKun Sun, YanFeng Chen, JinXiang Wang, Lei Sang, ChengFang Gao, XiPing Xie

**Affiliations:** 1 Institute of Animal Husbandry and Veterinary Medicine, Fujian Academy of Agricultural Sciences, Fuzhou, 350013 China; 2 Fujian Key Laboratory of Animal Genetics and Breeding, Fuzhou, 350013 China

**Keywords:** Minxinan black rabbits, Feed restriction, Growth and slaughter performance, Blood biochemistry, Intestinal morphology

## Abstract

Feed restriction after weaning is a common strategy used in commercial rabbit farms to improve feed efficiency, promote health, and reduce mortality. However, few studies have investigated the feed restrictions of Minxinan black rabbits (*Oryctolagus cuniculus*). Thus, the effects of feed restriction on growth and slaughter performance, intestinal morphology, and blood biochemical indices of Minxinan black rabbits were evaluated in this study. Rabbits in group A (control group) had ad libitum intake, while those in feed restriction groups (groups B, C, and D) were restricted to 80% of the average daily feed intake (ADFI) of group A the day before. The rabbits in group B were fed once per day at 8:00 am. Rabbits in groups C and D were fed twice per day at 8:00 am (50%) and 4:00 pm (50%) and 8:00 am (30%) and 4:00 pm (70%), respectively. The experimental period lasted for 8 weeks. Compared to that in group A, the diarrhea rate of group C was significantly decreased (*P* < 0.05), and the ADFI, feed conversion ratio, abdominal fat weight, abdominal fat rate, total protein, albumin, globulin, alanine aminotransferase (ALT), low-density lipoprotein, and intestinal crypt depth of all feed restriction groups were significantly reduced (*P* < 0.01). Feed conversion ratio in group D was significantly better than that in groups B and C (*P* < 0.05). The efficiency index (EI) of groups C and D was higher than that of groups A and B (*P* < 0.01). Triglyceride levels in groups C and D were significantly lower than those in group A. The villus length to crypt depth of the duodenum and jejunum in group D was significantly higher than that in group A (*P* < 0.01). In conclusion, the following parameters can be improved by feed restriction: feed conversion ratio, diarrhea rate, abdominal fat rate, serum ALT, lipid indices and intestinal health of Minxinan black rabbits, and the EI of the farm. Feeding twice per day, 30% at 8:00 am and 70% at 4:00 pm, had the best comprehensive effects.

## Introduction

Feed restriction programs have been used in post-weaning animals to improve feed efficiency and resistance to digestive system disorders, reducing carcass fat and production costs (Tumova et al. 2021). Studies on feed restriction in pigs (Le Floc'h et al. 2014), broilers (Van der Klein et al. 2017), and sheep (Lima et al. 2022) showed that animals can compensate the growth inhibition of feed restriction by improving feed efficiency. Moreover, feed restriction can be used to reduce morbidity and mortality in growing rabbits (Boisot et al. 2003; Gidenne et al. 2009; De Blas 2013), improve farm feed efficiency (Knudsen et al. 2014; Birolo et al. 2020a), and reduce nitrogen emissions and environmental pollution (Birolo et al. 2016; Birolo et al. 2017). It has been suggested to reduce rabbit feed intake by at least 20% of ad libitum to reduce post-weaning mortality and 30% to reduce morbidity (Gidenne et al. 2009; Gidenne and Feugier 2009). Time-based feed restriction significantly improves the feed conversion ratio of rabbits and promotes health (Birolo et al. 2020a, 2020b; Crespo et al. 2020).

The rabbit industry is crucial in China with highest breeding and export volume of rabbits in the world, and its rabbit meat export volume accounts for 27%, globally (Cullere and Dalle Zotte 2018). There is no uniform feed restriction program for rabbits on farms, and the effects of feed intake restriction and time-based feed restriction on local meat rabbits in China have not been reported. Minxinan black rabbits are a genetic resource of livestock and poultry in China. Therefore, determining the most suitable feed restriction strategy for rabbit farming is of practical significance.

Minxinan black rabbit is an excellent local breed in Fujian Province with better meat quality, strong resistance to adversity, and extensive adaptability. However, high diarrhea and mortality rates of rabbits after weaning have seriously impeded the development of the meat rabbit industry. Feed restriction plays an important role in reducing production costs and improving feed efficiency in many animals after weaning. However, feed restriction in Minxinan black rabbits has not been described.

Therefore, this study investigated the effects of four feeding methods on the growth and slaughter performance, blood biochemical indices, and intestinal structure of Minxinan black rabbits, and to provide a reference for selecting appropriate feeding methods for production.

## Materials and methods

### Animal resources, diets, and feeding programs

The experiments were conducted in the animal house of the Institute of Animal Husbandry and Veterinary Medicine, Fujian Academy of Agricultural Sciences, China. The size of each rabbit cage was 42 × 50 × 35 cm, with single cage feeding and ad libitum water. The rabbits were fed pellet diets with the following nutrient levels: DE 10.5–10.8 MJ/kg, CP 16.5–17.0%, and CF 14.5–16.0%. The experimental period was 61 days, including 5 days of pre-feeding period and 56 days of formal experimental period. A total of 144 Minxinan black rabbits from Longyan City Tongxian Rabbit Industry Development Co., LTD with healthy and similar body weights at the age of 30 days were randomly divided into four groups with three replicates per group and 12 rabbits per replicate (*n*=6 males; *n*=6 females). The control group (group A) had ad libitum intake. The other three groups (B, C, and D) were restricted by 80% of the average daily feed intake (ADFI) of group A the day before. The rabbits in group B were fed once per day 8:00 am. Rabbits in groups C and D were fed twice per day 8:00 am (50%) and 4:00 pm (50%) and 8:00 am (30%) and 4:00 pm (70%), respectively.

### Sample collection and indices determination

#### Growth performance

Individual live weight was recorded at the beginning (initial body weight, IBW) and end of the experiment (final body weight, FBW) in the morning before feeding. During the entire experiment, rabbit health was monitored daily. Diarrhea and mortality rates were recorded daily. Feed intake was measured daily by weighing the feed supplied and the refuse. The average daily gain (ADG), ADFI, feed conversion ratio, and efficiency index (EI) from weeks 5 to 13 were calculated.

$$\text{ADG }\left(\text{g}/\text{d}\right)=\left(\text{FBW}-\text{IBW}\right)\;/\text{test}\;\text{days};$$

$$\text{ADFI }\left(\text{g}/\text{d}\right)=\text{total}\;\text{feed}\;\text{intake}/\left(\text{test}\;\text{days}\times\text{number}\;\text{of}\;\text{test}\;\text{animals}\right);$$

$$\textrm{Feed}\ \textrm{conversion}\ \textrm{ratio}=\textrm{ADFI}/\textrm{ADG};$$

$$\text{Diarrhea}\;\text{rate }\left(\%\right)=\left(\text{number}\;\text{of}\;\text{rabbits}\;\text{suffering}\;\text{from}\;\text{diarrhea}/\text{total}\;\text{number}\;\text{of}\;\text{rabbits}\;\text{tested}\right)\times100;$$

$$\text{Mortality}\;\text{rate }\left(\%\right)=\left(\text{number}\;\text{of}\;\text{rabbit}\;\text{deaths}/\text{total}\;\text{number}\;\text{of}\;\text{rabbit}\text{s}\;\text{tested}\right)\times100;$$

$$\textrm{EI}=\left(\textrm{FBW}\times \textrm{survival}\ \textrm{rate}\right)/\left(\textrm{feed}\ \textrm{conversion}\ \textrm{ratio}\times \textrm{sale}\ \textrm{age}\right).$$

#### Slaughter performance

Eighteen healthy 13-week-old rabbits were recruited in each group, and six rabbits per replicate (*n* = 3 males and *n* = 3 females) were slaughtered. The slaughter process was conducted by the World Rabbit Science Association recommendations (Blasco A. and J. Ouhayoun 1996). Pre-slaughter body weight, eviscerated carcass weight, half eviscerated carcass weight, and abdominal fat weight were measured. Eviscerated carcass rate, half eviscerated carcass ratio, and abdominal fat rate were calculated. Eviscerated carcass weight was the carcass weight after slaughter, excluding blood, fur, head, tail, limbs (below wrist and hock), and viscera. Half eviscerated weight was the eviscerated carcass weight plus the heart, liver, kidney, and abdominal fat.

$$\text{Eviscerated}\;\text{carcass}\;\text{rate}\;\left(\%\right)=\left(\text{Eviscerated}\;\text{carcass}\;\text{weight}/\mathrm P\text{re}-\text{slaughter}\;\text{body}\;\text{weight}\right)\times100;$$

$$\text{Half}\;\text{eviscerated}\;\text{carcass}\;\text{rate}\;\left(\%\right)=\left(\mathrm H\text{alf}\;\text{eviscerated}\;\text{carcass}\;\text{weight}/\mathrm P\text{re}-\text{slaughter}\;\text{body}\;\text{weight}\right)\times100;$$

$$\text{Abdominal}\;\text{fat}\;\text{rate}\;\left(\%\right)=\left(\mathrm A\text{bdominal}\;\text{fat}\;\text{weight}/\mathrm P\text{re}-\text{slaughter}\;\text{body}\;\text{weight}\right)\times100.$$

#### Blood biochemical indices

Blood samples (2 mL) were obtained from the precaval vein of 18 rabbits per group (9 males and 9 females) at 13 weeks old. The samples were transferred to tubes and allowed to coagulate for 30 min before centrifugation at 3500 × *g* for 15 min. Isolated serum samples were collected and stored at −80 °C for further analysis. Blood biochemical indices were determined using a BECKMAN AU2700 automatic biochemical analyzer (Beckman, USA).

#### Intestinal morphology

After slaughter in the morning at the end of the experiment, 1.0–1.5 cm of intestinal tubes in the middle parts of the duodenum and jejunum were cut out. Next, the intestinal contents were washed away with plenty of normal saline and were moved into formalin fixed solution. After paraffin embedding, tissue sections, and hematoxylin and eosin (H&E) staining, the morphological structure of the intestine was examined using a microscope. Five complete villi from each section were selected using the Image-Pro Plus 6.0. Villi height and crypt depth were measured for five times respectively, and the average values were calculated.

#### Statistical analysis

Excel 2010 and SPSS 20.0 statistical software were used to process and analyze the data. One-way ANOVA and Tukey’s multiple comparison method were used to test the significance of differences between groups. *P* > 0.05 was not significant, *P* < 0.05 was significant, and *P* < 0.01 was extremely significant. 

## Results

### Growth performance and health status

There was no significant difference in IBW among all the groups (*P* > 0.05). From 5 to 13 weeks, the FBW, ADG, ADFI, and feed conversion ratio of the feed restriction groups were significantly lower than those of control group A (*P* < 0.01). Furthermore, from 5 to 13 weeks, the diarrhea rate in group A was the highest (25.00%), followed by groups B (19.44%), C (8.33%), and D (8.33%), which was significantly lower than that in group A (*P*< 0.05). There was no significant difference in mortality rate among all the groups (*P* > 0.05). The EI of groups C and D were higher than that of groups A and B (*P* < 0.01) (Table 1).

**Table 1:** Effects of feeding methods on growth performance of Minxinan black rabbits

Trait	Group A	Group B	Group C	Group D	SEM	*P* value
IBW (g)	537.56	545.35	533.94	534.33	7.51	0.692
FBW (g)	1737.21^Aa^	1641.42^Bb^	1638.42^Bb^	1654.22^Bb^	14.06	<0.001
ADG (g/d)	21.41^Aa^	19.58^Bb^	19.72^Bb^	20.00^Bb^	0.25	<0.001
ADFI (g/d)	107.07^Aa^	86.49^Bb^	86.61^Bb^	85.54^Bb^	0.49	<0.001
Feed conversion ratio	5.00^Aa^	4.42^Bb^	4.39^Bb^	4.28^Bb^	0.02	<0.001
Diarrhea rate (%)	25.00^Aa^	19.44^Aab^	8.33^Ab^	8.33^Ab^	3.10	0.029
Mortality rate (%)	5.56	8.33	2.78	5.56	2.09	0.487
EI	360.45^Bb^	373.85^Bb^	398.77^Aa^	401.41^Aa^	2.91	<0.001

*SEM*, standard error of the mean; *IBW*, initial body weight; *FBW*, final body weight; *ADG*, average daily gain; *ADFI*, average daily feed intake; *EI*, efficiency index. In the same line, values with no letter or the same letter superscripts mean no significant difference (*P* > 0.05), while with different small letter superscripts mean significant difference (*P* < 0.05), and with different capital letter superscripts mean significant difference (*P* < 0.01) by the Tukey’s test

### Slaughter traits

The pre-slaughter weight, eviscerated carcass and half eviscerated carcass weights, abdominal fat weight, and abdominal fat rate of feed restriction groups B, C, and D were significantly lower than those of group A (*P* < 0.01); however, no significant differences in eviscerated carcass rate and half eviscerated carcass rate were observed among all the groups (*P* > 0.05) (Table 2).

**Table 2:** Effects of feeding methods on slaughter performance of Minxinan black rabbits

Trait	Group A	Group B	Group C	Group D	SEM	*P* value
Pre-slaughter weight (g)	1731.93^Aa^	1640.97^Bb^	1634.64^Bb^	1643.56^Bb^	9.36	<0.001
Eviscerated carcass weight (g)	877.55^Aa^	848.90^ABab^	838.75^Bb^	833.69^Bb^	7.76	<0.001
Eviscerated carcass rate (%)	50.66	51.74	51.31	50.72	0.36	0.124
Half eviscerated carcass weight (g)	978.39^Aa^	925.50^Bb^	917.75^Bb^	912.44^Bb^	7.94	<0.001
Half eviscerated carcass rate (%)	56.48	56.41	56.14	55.51	0.36	0.231
Abdominal fat weight (g)	21.58^Aa^	16.03^Bb^	16.08^Bb^	16.03^Bb^	0.24	<0.001
Abdominal fat rate (%)	1.24^Aa^	0.98^Bb^	0.98^Bb^	0.98^Bb^	0.01	<0.001

*SEM*, standard error of the mean. In the same line, values with no letter or the same letter superscripts mean no significant difference (*P* > 0.05), while with different small letter superscripts mean significant difference (*P* < 0.05), and with different capital letter superscripts mean significant difference (*P* < 0.01) by the Tukey’s test

### Blood biochemical indices

As shown in Table 3, the contents of total protein (TP), albumin (ALB), globulin (GLB), and alanine aminotransferase (ALT) determined for group A were significantly higher than those for the feed restriction groups (*P* < 0.01). There were no significant differences in glutamyltranspeptidase, total cholesterol (CHOL), and high-density lipoprotein (HDL) levels among the groups (*P* > 0.05). Triglycerides (TG) of group A were significantly higher than that of group B (0.96 vs. 0.82 mmol/L, *P* < 0.05) and extremely significantly higher than that of groups C and D (0.96 vs. 0.63 and 0.54 mmol/L, *P* < 0.01). The low-density lipoprotein (LDL) levels in group A were significantly higher than those in the feed restriction groups (*P* < 0.01), and there was no significant difference among the feed restriction groups (*P* > 0.05).

**Table 3:** Effects of feeding methods on blood biochemical indexes of Minxinan black rabbits

Trait	Group A	Group B	Group C	Group D	SEM	*P* value
TP (g/L)	56.47^Aa^	38.29^Cc^	42.39^Bb^	39.97^BCbc^	0.76	<0.001
ALB (g/L)	34.50^Aa^	26.50^Bb^	27.97^Bb^	26.93^Bb^	0.39	<0.001
GLB (g/L)	21.98^Aa^	11.79^Cc^	14.41^Bb^	13.04^BCbc^	0.46	<0.001
ALT (U/L)	63.78^Aa^	48.14^Bb^	47.63^Bb^	45.80^Bb^	2.23	<0.001
GGT (U/L)	7.53	7.02	6.64	7.13	0.32	0.270
CHOL (mmol/L)	1.49	1.32	1.44	1.50	0.12	0.649
TG (mmol/L)	0.96^Aa^	0.82^Ab^	0.63^Bc^	0.54^Bc^	0.04	<0.001
HDL (mmol/L)	0.68	0.71	0.72	0.73	0.03	0.558
LDL (mmol/L)	0.47^Aa^	0.42^Bb^	0.41^Bb^	0.39^Bb^	0.04	<0.001

*SEM*, standard error of the mean. In the same line, values with no letter or the same letter superscripts mean no significant difference (*P* > 0.05), while with different small letter superscripts mean significant difference (*P*<0.05), and with different capital letter superscripts mean significant difference (*P* < 0.01) by the Tukey’s test

### Intestinal morphology

**Fig. 1:**
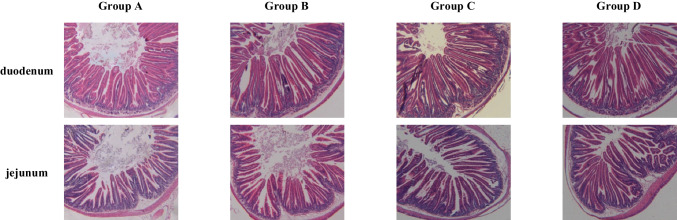
Morphology of intestinal tissue sections of Minxinan black rabbits (100×)

The villus length of the duodenum in group A was significantly shorter than that in groups C and D (*P* < 0.01). The villus length of the jejunum in group A was shorter than that in group C (*P*<0.05). The crypt depth of the jejunum in group A was significantly higher than that in the feed restriction group (*P* < 0.01), and that of group B was significantly lower than that of group C (*P* < 0.05). There was no significant difference in duodenal crypt depth between all the groups (*P* > 0.05). The ratio of villus length to crypt depth (V/C) of the duodenum and jejunum in group D was significantly higher than that in group A (*P* < 0.01) (Table 4 and Fig. 1).

**Table 4:** Effects of feeding methods on intestinal morphology of Minxinan black rabbits

Trait	Tissue	Group A	Group B	Group C	Group D	SEM	*P* value
Villus length (μm)	Duodenum	895.41^Cc^	932.93^BCbc^	995.66^ABab^	1048.54^Aa^	15.62	<0.001
	Jejunum	633.58^ABbc^	595.61^Bc^	714.75^Aa^	705.82^Aab^	19.84	<0.001
Crypt depth (μm)	Duodenum	254.91	217.74	227.8	209.89	13.22	0.103
	Jejunum	177.70^Aa^	106.65^Cc^	139.23^Bb^	115.38^BCc^	5.72	<0.001
V/C	Duodenum	3.88^Bb^	4.88^ABab^	4.67^ABab^	5.57^Aa^	0.31	0.006
	Jejunum	3.82^Bb^	5.87^Aa^	5.57^Aa^	6.38^Aa^	0.28	<0.001

*SEM*, standard error of the mean; *V/C*, villus length/crypt depth. In the same line, values with no letter or the same letter superscripts mean no significant difference (*P* > 0.05), while with different small letter superscripts mean significant difference (*P* < 0.05), and with different capital letter superscripts mean significant difference (*P* < 0.01) by the Tukey’s test

## Discussion

Studies have shown that diet controlled at 95% ad libitum feed intake does not affect the growth and slaughter performances of growing rabbits, and 85% do not affect growth rate, and that the feed conversion ratio remains unchanged (Romero et al. 2010). The diarrhea rate and mortality rate of post-weaning rabbits can be reduced only when the intake is controlled at less than 80% of ad libitum intake, with little impact on growth and slaughter performances (Gidenne and Feugier 2009). However, a protective effect of feed restriction on intestinal health has been found, which disappears after the resumption of ad libitum feeding (Knudsen et al. 2014; Alabiso et al. 2017). The sudden resumption of ad libitum feeding from feed restriction usually leads to a peak in feed intake and a high-risk of digestive tract disease (Akbarian et al. 2014; Knudsen et al. 2014). The method of gradual transition from feed restriction to ad libitum feeding can prevent excessive feeding, and wasted feed, and the intake peak and excessive consumption of the diet in the fattening stage (Birolo et al. 2020b), but the method of gradual transition is immature, making it difficult to apply.

In this report, from 5 to 13 weeks, control group A had ad libitum intake, while the other three groups were restricted 80% of ad libitum intake in group A the day before. There was no significant difference in mortality rate among all the groups (*P* > 0.05). The diarrhea rate of group A was the highest (25%) among all the groups and significantly higher than that of group C (*P* < 0.05), and these findings are consistent with previous report (Gidenne and Feugier 2009) showing reduced diarrhea rate upon feed restriction. Notably, some studies have shown that restricted feeding in post-weaning rabbits can reduce morbidity and mortality (Gidenne and Feugier 2009; Knudsen et al. 2014; Birolo et al. 2016; Knudsen et al. 2017), but other studies have shown that feed restriction tended to increase rabbit mortality rate (Birolo et al. 2020a). The explanation for differences among studies remains unclear, but a combination of factors, such as diet, farm health status, methods of feed restriction used, or housing type, are possible reasons.

The FBW, ADFI, ADG, and feed conversion ratio of feed restriction groups B, C, and D were significantly lower than those of group A (*P* < 0.01). Among the feed restriction groups, the EI of group D showed the highest. Feed conversion was improved significantly in all treatments B, C, and D. These results indicate that a feed restriction of 80% ad libitum intake can reduce the diarrhea rate and increase the feed conversion ratio; the growth rate was also affected, which could be compensated by reducing the diarrhea rate. EI is a comprehensive measure of rabbit production, reflecting various indicators of a rabbit flock, namely body weight, survival rate, feed conversion ratio, and production management. The EI of groups C and D was higher than that of groups A and B (*P* < 0.01).

Slaughter performance reflects the deposition and distribution of nutrients in different parts of meat rabbits. Eviscerated carcass weight, eviscerated carcass rate, half eviscerated carcass weight, and half eviscerated carcass rate are important indicators for measuring the slaughter performance of meat rabbits, and these indicators are closely related to the dietary nutrition level. In this trial, the pre-slaughter weight, eviscerated carcass weight, half carcass weight, abdominal fat weight, and abdominal fat percentage in the restricted feeding groups were significantly lower than those in group A (*P* < 0.01), but eviscerated and half eviscerated carcass rate were not significantly different from those in group A (*P* > 0.05). These results may be attributed to the relatively balanced development rate of all parts of the Minxinan black rabbit body during the growth stage (from weaning to market). These findings suggest that feed restriction of 80% ad libitum intake by using different methods does not affect the slaughter rate, which is consistent with precious findings (Birolo et al. 2020a). Similarly, Knudsen et al. found that the slaughter rate of growing rabbits was significantly reduced when growing rabbits were fed at 75% ad libitum intake for 4 weeks (Knudsen et al. 2017). By contrast, feed restriction significantly reduced the percentage of perirenal fat (Tumova et al. 2021; Tůmová et al. 2021). In the current study, the abdominal fat and abdominal fat percentages in the feed restriction groups were significantly lower than those in the ad libitum feeding group A.

The gut is an important defense barrier in humans and animals (Yang et al. 2019). In the small intestine, digestion and absorption occur, and the integrity of its mucosal morphology reflects the digestion and absorption of the diet. Villi length and crypt depth are the most direct indicators of the morphological integrity of small intestinal mucosa. The longer the villi in the small intestine, the larger the absorption area and the stronger the absorption and utilization of nutrients. The intestinal crypt depth reflects the maturity rate of epithelial cells. The shallower the crypt, the better the digestion and absorption. In this experiment, the crypt depth of the jejunum in the feed restriction groups was significantly lower than that in group A (*P* < 0.01). The villus length of the duodenum was significantly longer in group D than that in group A. The V/C of the duodenum and jejunum in group D was significantly higher than that in group A (*P* < 0.01). The results showed that group D exhibited the best intestinal improvement and was beneficial for improving the absorption and utilization of feed nutrients.

Serum biochemical indices can reflect the metabolic status of animal physiology (Ewuola 2008). Serum TP and ALB levels mainly reflect the absorption, synthesis, and decomposition of proteins. Serum GLB is a mixture of a various proteins, including immunoglobulins, and complements, and a various glycoproteins. In this experiment, the contents of TP, ALB, and GLB in group A were significantly higher than those in the feed restriction groups (*P* < 0.01), indicating that feed restriction of 80% ad libitum intake reduced protein absorption. Serum ALT level is an important indicator of liver function. Under normal physiological conditions, ALT activity is maintained at relatively low levels. Only when permeability increases induced by cell damaged will increase ALT activity, and then enter the blood, increasing the content of ALT in serum. The results of this study showed that ALT levels in the feed restriction groups were significantly lower than those in group A (*P* < 0.01); therefore, feed restriction reduced ALT levels and had a protective effect on liver function. Similar results were reported in a study on fattening pigs (Batorek et al. 2012). Levels of CHOL, TG, HDL, and LDL in the serum can reflect the lipid metabolism of the body. The decrease in CHOL, TG, and LDL levels benefits hyperlipidemia prevention. There were no significant differences in CHOL and HDL levels among the groups (*P* > 0.05). TG in groups B, C, and D was significantly lower than that in group A (*P* < 0.05), which may be because the energy intake of rabbits in the feed restriction groups could not fulfill the needs of the body, leading to a decrease in the metabolic rate, and the serum TG needed to be transported to various tissues and organs for the oxidation of energy. In this study, LDL in the feed restriction groups was significantly lower than that in group A (*P* < 0.01), indicating that feed restriction reduced serum LDL content, which is consistent with the results of precious studies (Xu et al. 2017).

## Conclusion

The feed restriction of 80% ad libitum intake improved the intestinal morphology of Minxinan black rabbits; promoted intestinal health, increased the EI; decreased the serum lipid content, feed conversion ratio, and diarrhea rate; and the feed restriction method of feeding twice per day (30% at 08:00 am and 70% at 4:00 pm) had the best comprehensive effects.

## Data Availability

The data that support the findings of this study are available from the corresponding author upon reasonable request.
